# Symptom burden, lung function, exercise tolerance and inflammation in patients post COVID-19: results from the prospective COVID-19 Chronic Morbidity (CCHROMO) study

**DOI:** 10.1186/s12890-026-04365-1

**Published:** 2026-06-17

**Authors:** Carla Bellinghausen, Achim Grünewaldt, Tara Azem, Cornelius Lask, Wenhan Du, Alexander Seeger, Eva Herrmann, Ralf Schubert, Julia Hafner, Maria J. G. T. Vehreschild, Tobias Manuel Appel, Gernot Rohde

**Affiliations:** 1 Department of Respiratory Medicine, Goethe University Frankfurt, University Hospital, Medical Clinic 1, Frankfurt am Main, Germany; 2 Institute of Biostatistics and Mathematical Modelling, Goethe University Frankfurt, Frankfurt am Main, Germany; 3 Department of Pediatrics, Division of Pneumology, Allergology, Infectious diseases, and Gastroenterology, Goethe University Frankfurt, University Hospital, Frankfurt am Main, Germany; 4 Goethe University Frankfurt, University Hospital Frankfurt, Medical Clinic 2, Infectious Diseases, Frankfurt am Main, Germany; 5 Fraunhofer Institute for Translational Medicine and Pharmacology ITMP, Frankfurt am Main, Germany; 6 Department of Respiratory, Intensive Care and Sleep Medicine, Faculty of Medicine, Marburg-University, Member of the German Center for Lung Research (DZL), Baldingerstraße, Marburg, 35033 Germany; 7 CAPNETZ Stiftung, Hannover, Germany

**Keywords:** post acute infection syndrome, adults, outpatients

## Abstract

**Background:**

Post-COVID syndrome is common and associated with substantial morbidity, yet its pathophysiology and predictors of disease course remain incompletely understood. This study aimed to quantify symptom burden in patients attending a post-COVID outpatient clinic and to evaluate associations with pulmonary function parameters, exercise capacity, and circulating biomarkers.

**Methods:**

The prospective CCHROMO cohort collects longitudinal clinical data, detailed pulmonary function testing, and blood samples from patients with persistent symptoms following SARS-CoV-2 infection, with planned follow-up of up to three years. We present an interim analysis of 253 patients contributing 561 study visits (median follow-up 273 days after COVID-19 diagnosis; interquartile range [IQR] 177–332). Assessments included standardized symptom questionnaires, spirometry, inspiratory muscle strength testing, cardiopulmonary exercise testing (CPET), and serum biomarker measurements reflecting inflammation as well as epithelial and endothelial injury.

**Results:**

At study entry, the most prevalent symptoms were fatigue (84.6% reporting at least moderate severity), dyspnoea (70.3%), headache (69.8%), and neurocognitive impairment (66.4%). Spirometric values were within normal ranges in most participants. In contrast, reduced inspiratory muscle strength was observed in 54.8% of visits, and impaired exercise capacity on CPET in 28.8%. Correlations between symptom severity, pulmonary function, CPET parameters, and circulating biomarkers were weak. Serum club cell protein-16 (CC-16) levels were lower in patients with severe fatigue (median [IQR] 6.6 [4.7–8.4] ng/mL) compared with those without fatigue (7.6 [6.3–10.1] ng/mL; *p* = 0.039).

**Conclusions:**

Patients with post-COVID syndrome show a high and persistent symptom burden, frequently accompanied by reduced inspiratory muscle strength and impaired exercise capacity despite preserved spirometry. Associations between symptoms, physiological measures, and biomarkers were weak and suggest heterogenous post-COVID manifestations.

**Trial registration:**

German Clinical Trials Register (DRKS00022475, Date of Registration 2020-07-27).

**Supplementary Information:**

The online version contains supplementary material available at 10.1186/s12890-026-04365-1.

## Summary

What is already known about this topic:

A substantial proportion of individuals following SARS-CoV-2 infection develop persistent symptoms, including fatigue, dyspnoea, and neurocognitive impairment. The underlying biological mechanisms and reliable predictors of disease course remain poorly defined.

What this study adds:

This study provides integrated longitudinal data combining standardized symptom assessments, pulmonary function testing, cardiopulmonary exercise testing, and circulating biomarkers in a large post-COVID outpatient cohort. Reduced inspiratory muscle strength and impaired exercise capacity were observed in approximately half and one third of visits, respectively, despite preserved spirometry. Serum concentrations of club cell protein-16 (CC-16), a marker of epithelial integrity with reported anti-inflammatory properties, were lower in patients with severe fatigue. These findings highlight the clinical impact and mark heterogeneity of post-COVID manifestations.

How this study might affect research, practice or policy:

The dissociation between symptom burden and conventional pulmonary function measures indicates that routine spirometry alone may be insufficient to characterize post-COVID morbidity. Prolonged recovery times and subtle functional limitations should be considered in outpatient management and follow-up strategies. Biomarker profiling, including CC-16, may contribute to future phenotyping approaches, but requires validation in larger cohorts and in other post-viral syndromes before clinical implementation.

## Introduction

Persistent health problems following SARS-CoV-2 infection have emerged as a major public health problem following the COVID-19 pandemic. Currently, there are several approaches to defining long COVID and the post COVID-19 syndrome. The National Institute for Health and Care Excellence (NICE) defines post-COVID-19 syndrome as “persisting or developing signs and symptoms during or after a COVID-19 infection that continue for more than 12 weeks” (National Institute for Health and Care Excellence (NICE), 2020). According to the 2022 World Health Organization (WHO)-guided Delphi process definition, post COVID-19 condition can be diagnosed in patients with persistent symptoms three months after acute infection, that last at least two months and that cannot be explained by an alternative diagnosis [1].

Estimates of the prevalence of post COVID-19 syndrome vary substantially, even among meta analyses, ranging from approximately 6 to 42% [2–4]. Different definitions of long COVID or post COVID-19 syndrome, regional differences, and differences in the study population, amongst other things, contribute to this heterogeneity. Nevertheless, the substantial impact of post COVID-19 syndrome on public health is beyond doubt. Post COVID-19 syndrome presents as multisystemic disease with broad clinical manifestations ranging from respiratory symptoms and cardiac impairment to neurological and psychiatric symptoms [5, 6]. The majority of patients with post COVID-19 syndrome are impaired by shortness of breath, chest tightness, sleep disturbances and impaired concentration and fatigue [7].

Enormous efforts are being made to understand the underlying mechanisms to identify treatment options. At the moment, treatment options encompass mainly concepts for rehabilitation [8, 9]. Numerous factors have been suggested to play a role in the pathogenesis of post-COVID-19 syndrome, including dysregulated inflammation [10–12], the generation of autoantibodies [13, 14], virus persistence [15], reactivation of latent viruses [16], fibrotic changes in the lungs [17], and neuronal and glial dysregulation [18]. Nevertheless, to date, the pathomechanisms of post COVID-19 syndrome remain unclear.

Recently, an increasing number of prospective post COVID-19 observational studies has been published. Some of these studies have explored the use of cardiopulmonary exercise testing (CPET) for assessing exercise capacity [19–24]. Although these publications describe a frequent limitation in exercise capacity in patients with post COVID-19 syndrome, there appears to be no correlation with the severity of the acute illness [20, 25].

To our knowledge no studies have correlated these findings with potential underlying factors of impaired exercise capacity. In our study, we evaluated the association between reduced oxygen uptake and underlying factors such as impaired respiratory muscle strength, as measured by maximum inspiratory pressure, MIP, and current inflammatory status.

Here, we present an interim analysis of data from a prospective observational study aimed at characterizing symptom burden, lung function, CPET results and circulating markers of inflammation and pulmonary damage in patients after acute SARS-CoV2 infection (COVID-19 Chronic Morbidity [CCHROMO] study, DRKS00022475).

## Methods

### Study design

We here present data of a first interim analysis of an ongoing, prospective observational study (COVID-19 Chronic Morbidity Study, CCHROMO). Participants were recruited among patients attending the outpatient clinics of the Departments of Respiratory Medicine and Infectious Diseases at University Hospital Frankfurt due to persistent complaints following COVID-19. Inclusion criteria were age ≥ 18 years and evidence of recent infection with SARS-CoV-2. At the time of initiation of the study, no generally accepted definition of post COVID-19 syndrome existed. Therefore, the study was open to all patients with persistent or new onset of symptoms after a SARS-CoV-2 infection presenting to our outpatient service because of persisting symptoms 4 weeks or more after infection with SARS-CoV-2. Pregnant women were excluded from the study.

As part of the study, participants can be followed up for up to three years after infection. During this period, data are recorded prospectively during all visits in the outpatient clinic, which usually take place in 3-month intervals as long as symptoms persist. In this analysis, we present data on lung function, symptoms and laboratory data of patients recruited between September 2020 and July 2021, with follow up data until December 2021. The maximum time after infection included in the present data set is 543 days.

### Statement on patient and public involvement

Patients or patient organisation were not involved in the design of the study.

### Ethics approval and consent to participate section

The study was designed and conducted according to the requirements of the Declaration of Helsinki. The protocol was approved by the institutional review board of the medical faculty of Goethe University Frankfurt (20–748) and registered in the German Clinical Trials Register (DRKS00022475, Date of Registration 2020-07-27). All participants provided written informed consent prior to inclusion in the study.

### Symptom scores and questionnaires

Symptoms were recorded using an 18-item questionnaire originally developed for pneumonia-associated symptoms [26]. At the time of study design, questionnaires specifically designed for the evaluation of long COVID-19 / post COVID-Syndrome were not available. Fatigue was assessed using the Fatigue Assessment Scale (FAS), a 10-item questionnaire which evaluates self-reported symptoms of chronic fatigue. FAS scores ≥ 22 were considered to be indicative of chronic fatigue, FAS scores ≥ 35 were considered as indicating extreme fatigue [27]. Any information about former diseases were taken by patient’s history.

### Lung function measurements and Cardiopulmonary Exercise Testing (CPET)

Patients received standardized lung function tests including spirometry, body plethysmography, CO-diffusion test and measurement of inspiratory muscle strength (body plethysmograph type MasterScreen, Carefusion, software Sentry Suite V3.10.5). The procedure was performed in accordance with recommendations of the German Respiratory Society [28–30]. Baseline parameters of pulmonary function testing prior to study enrolment were not considered. Based on the patient’s symptoms, such as persistent dyspnoea or tightness in the chest, the decision to conduct CPET was at the discretion of the attending physician. CPET was performed via bicycle-test (Ergoline GmbH, “Viasprint 200”, CareFusion Germany “Vyntus-CPX”). Patients underwent a ramped 10-minute test protocol with symptom limitation. The minimal workload at the beginning was 25 W and was adapted depending on the patient reported fitness. Termination criteria were extensively deranged blood pressure or high-grade arrhythmias. All sessions were terminated with e cool down of regularly at least three minutes. The examinations were monitored by a physician, who was allowed to adapt the workload and decided about the termination of the exercise test.

### Measurement of cytokines and epithelial and endothelial markers

Interleukin (IL-)8 was measured from serum samples by cytometric bead array (CBA, BD Biosciences, Enhanced Sensitivity Flex Sets). Measurement was performed as multiplex measurement together with IL-1β and IL-10. Since measurements for IL-1β and IL-10 were below the range for reliable quantification in > 75% of samples, results for these cytokines are not presented. Measurements were performed as per the manufacturer’s instructions on a BD FACSVerse Cell Analyzer. Concentrations of all remaining markers analysed were determined by ELISA. Analytes included transforming growth factor β (TGF- β, ThermoFisher Scientific); club cell protein (CC-) 16 and surfactant protein D (SP-D, both from BioVendor); and IL-33, Angiopoietin-2, and vascular cell adhesion molecule (VCAM-) 1 (all from Sigma Aldrich).

In pilot experiments, we additionally performed measurements of IL-6 (CBA, BD Biosciences), IL-17 (ELISA, R&D Systems), IL-1RA (ELISA; abcam), interferon (IFN-)γ (CBA, BD Biosciences), Tumour necrosis factor (TNF-)α (CBA, BD Biosciences) and intercellular adhesion molecule (ICAM-)1 (ELISA, abcam) on randomly selected subsets of serum samples. Since concentrations were below the detection or quantification limit in more than 75% of samples, these results were not considered in further analyses.

### Statistical analysis

Data were analysed using IBM SPSS Statistics v29 and RStudio running R v4.3.1 [31] and tidyverse v2.0.0 [32]. Data are presented as n (% of available data) or median (interquartile range, IQR). Linear mixed models were computed using R packages lme4 v1.1-35.2 [33] and lmerTest v3.1-3 [34], using patient identifier as random effects variable. Concentrations of serum proteins were log10 transformed for statistical analysis. Results of linear mixed models were visualised using sjPlot v2.8.15 [35]. All other graphs were created using packages ggplot2 v3.5.0 [36], ggpubr v0.6.0 [37] and ggcorrplot v0.1.4.1 [38].

## Results

### Study population

The analyses presented here include data of patients recruited between September 2020 and July 2021 among patients attending the outpatient clinic of the University Hospital Frankfurt following COVID-19. Follow up data of these patients until December 2021 were included in the analysis. In total, data of 253 patients at 561 visits were included in our analyses, with a median number of two visits per patient.

Sociodemographic data are summarised in Table 1. The median age of patients at inclusion was 48 years (interquartile range [IQR]: 37–56). 164/253 (64.8%) of patients were female. At inclusion, the median body mass index (BMI) was 25.5 (IQR: 22.8–28.7) kg/m^2^. Most participants were never smokers (59.1%) or former smokers (35.3%). The most common pre-existing respiratory co-morbidity recorded was bronchial asthma (12.5%), whereas COPD and interstitial lung diseases were uncommon in this group (< 1% each).

**Table 1:** Sociodemographic data and severity of acute COVID-19

Sociodemographic data	*N* available	median (IQR) or *n* (% of data available)
Age [years]	253	48 (37–56)
Female gender	253	164 (64.8%)
BMI [kg·m^-2^]	235	25.5 (22.8–28.7)
Smoking	252	
*Never smokers*		149 (59.1%)
* Active smokers*		14 (5.6%)
*Former smokers*		89 (35.3%)
Co-morbidities	248	
*None*		193 (77.8%)
*Bronchial asthma*		31 (12.5%)
*COPD*		2 (0.8%)
*Interstitial lung disease*		2 (0.8%)
*Other*		20 (8.1%)
*Course of acute COVID-19*		
Hospitalisation	251	37 (14.7%)
*Intensive care*		8 (21.6% of hospitalised patients)
Ventilation	249	
*Invasive*		2 (0.8%)
*Non-invasive*		6 (2.4%)
Extracorporeal Membrane Oxygenation (ECMO)	249	0 (0%)
Severity of acute COVID-19^¶^	251	
*1 – Mild disease*		84 (33.5%)
*2 – Pneumonia*		144 (57.4%)
*3 – Severe pneumonia*		21 (8.4%)
*4 – ARDS*,* sepsis*,* septic shock*		2 (0.8%)

*BMI* body mass index, *COPD* chronic obstructive pulmonary disease, *ARDS* acute respiratory distress syndrome, *IQR* interquartile range

^¶^ based on “COVID-19 Clinical Management”, WHO [39]

Initial infections with SARS-CoV-2 dated from the beginning of the pandemic until May 2021, corresponding to the first three pandemic waves in Germany [40]. Most patients of our cohort had experienced a mild to moderate course of acute COVID-19, with 37 (14.7%) requiring hospitalisation during the acute phase of the disease. Of these, 8 patients (21.6% of hospitalised patients) were treated in an intensive care setting. Mechanical ventilation was required in 8 (3.2%) of cases (non-invasive ventilation: 6 cases [2.4%], invasive ventilation: 2 cases [0.8%]). The median time between diagnosis of COVID-19 and first presentation in the outpatient clinic was 106 days (IQR 79–180 days).

### Symptom burden

**Fig. 1:**
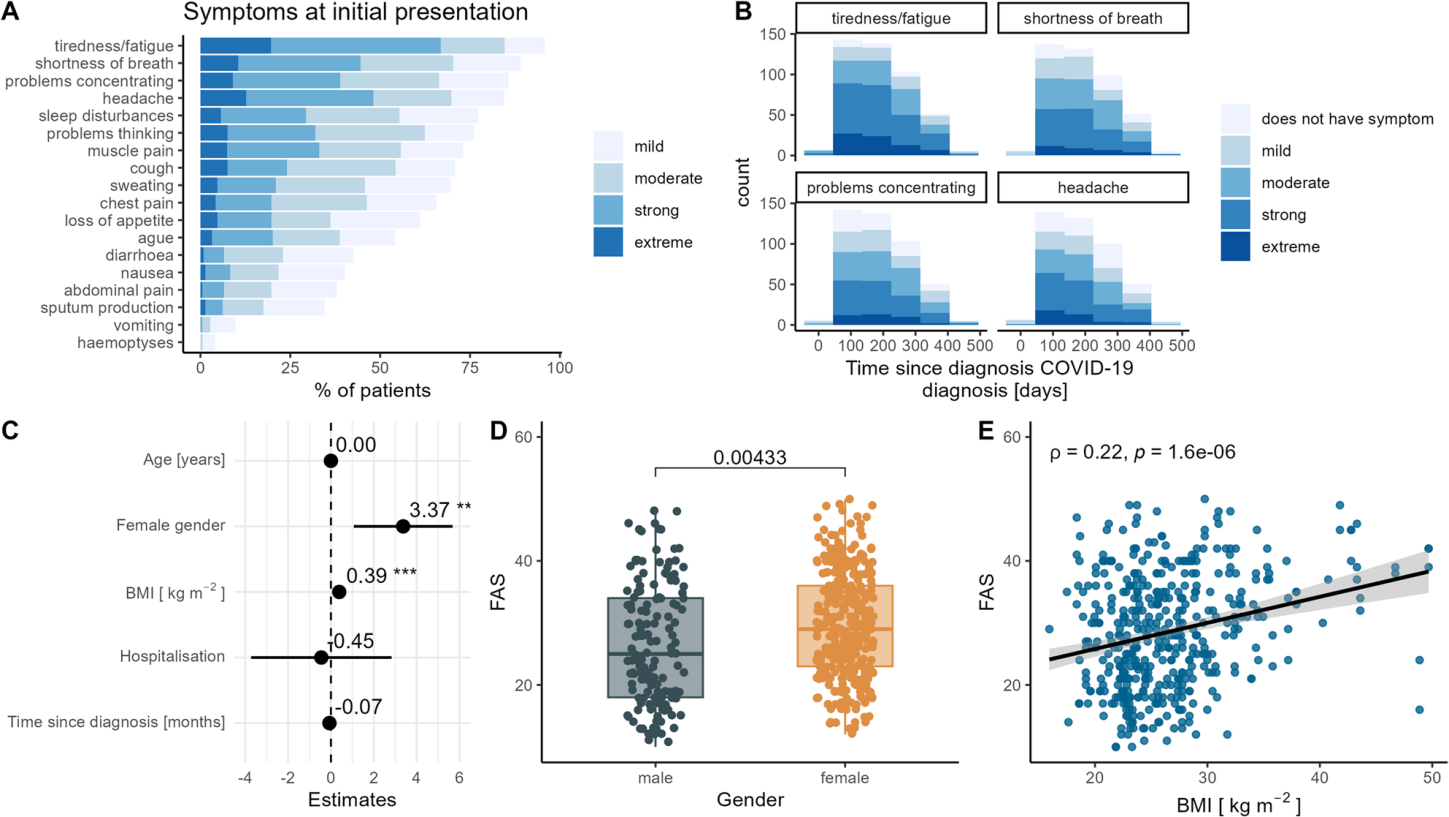
Symptom burden and predictors of fatigue. Occurrence and severity of symptoms were recorded using an 18-item questionnaire related to assessment of pneumonia symptoms (CAP18) and the Fatigue Assessment Scale (FAS). **A** Occurrence and severity of individual symptoms at initial presentation, based on CAP18 questionnaire. Data represent percentage of available symptom questionnaires. **B** Occurrence and severity of symptoms during all recorded visits in relation to time since diagnosis of acute COVID-19. **C** Results of linear mixed models for estimated effects of demographics and disease severity on FAS scores (unadjusted univariable analysis, patient identifiers were used as random effects). **D** FAS scores in men and women across all visits analysed. Statistical analysis was performed using linear mixed models with gender as fixed effect and patient identifier as random effects. **E** Correlation of BMI and FAS scores across all visits analysed (Spearman-Rank correlation). Abbreviations: BMI: body mass index; FAS: fatigue assessment scale

Complete CAP18 symptom questionnaires were available from 151 /253 patients at initial presentation, another 71 had partially completed the questionnaire. Upon initial presentation to the outpatient clinic, the most common symptoms (independent of their severity) were fatigue (205/214 [95.8%] of patients for whom answers to this question in the symptom questionnaire were available), shortness of breath (186/209 [89.0%]), concentration problems (181/211 [85.8%]), and headache (179/212 [84.4%]). A large proportion of patients stated that these symptoms affected them “strongly” or “extremely” (Fig. 1A). Considering data of all recorded visits, the proportion of patients who reported to be affected strongly or extremely by the most common symptoms remained high regardless of time passed since initial infection (Fig. 1B).

Fatigue was assessed in more detail using the Fatigue Assessment Scale (FAS). Based on this 10-item, self-administered scoring questionnaire, patients fulfilled the criteria for fatigue (FAS score ≥ 22) during 332/453 [73.3%] of visits with available data. During 132/453 [29.1%] of all visits, criteria for extreme fatigue were fulfilled (FAS score ≥ 35). Effects of demographic factors (age, gender, BMI) and factors related to acute disease (hospitalisation, time since diagnosis of acute COVID-19) on FAS scores were assessed individually using linear mixed models (Fig. 1C). Among the variables tested, only gender and BMI had a significant influence on FAS scores, with median FAS being significantly higher in female participants than in males (median FAS [IQR]: 29 [23–36] vs. 25 [18–34], *p* = 0.004), and BMI being positively correlated with FAS scores. Gender and BMI as predictors for symptom severity were not unique for fatigue but were also found to be significant predictors for overall symptom scores as assessed with the CAP18 questionnaire (estimated fixed effect of female gender + 6.202 points, *p* < 0.001; estimated fixed effects of BMI + 0.4185 points per kg/m^2^, *p* = 0.0026).

### Spirometry and blood gas analysis

Table 2 and Supplementary Table S1 summarize the results of lung function tests. Lung function parameters, including forced expiratory volume in one second (FEV_1_), forced vital capacity (FVC), peak expiratory flow (PEF), functional residual capacity (FRC), total lung capacity (TLC), and residual volume (RV) were within a physiological range during approximately 80 to 90% of all visits. Median values were ≥ 95% of predicted normal for all these parameters (Table 2, Supplementary Table S1). The median single breath measurement of DLCO was 75% of the predicted value. 66.4% of the results were outside the reference range (see Supplementary table S1). The median of transfer factor (KCOcSB) measurements was 88% of predicted (IQR 81–96%). In 98 visits (18.0% of visits with available data from blood gas analysis), pO_2_ levels below reference values were measured, in 36.3% pCO_2_ was below reference values. Hypercapnia was not observed. Lung function parameters showed very little to no correlation with symptom severity as assessed in standardised questionnaires (Supplementary Figure S1).

**Table 2:** Lung function, inspiratory muscle strength and CPET across all visits

Variable	*N* available	Median (IQR)	Outside reference range^#^ *n* (%)
*Lung function*			
FEV_1_ [% predicted]	558	97 (87–106)	72 (12.9%)
FVC [% predicted]	558	95 (85–103)	78 (14.0%)
FEV_1_ / VC [%]	558	83 (78–86)	18 (3.2%)
TLC [% predicted]	543	101 (92–110)	42 (7.7%)
KCOcSB [% predicted]	533	88 (81–96)	102 (19.1%)
*Blood gas analysis*			
pH	543	7.42 (7.41–7.44)	55 (10.1%)
pCO_2_ [mmHg]	545	36.3 (33.9–38.7)	197 (36.3%)
pO_2_ [mmHg]	546	94.0 (86.1–101.0)	98 (18.0%)
SaO_2_ [%]	546	97.9 (97.0–98.0)	22 (4.0%)
*Inspiratory muscle strength*			
p0.1 [kPa]	498	0.20 (0.13–0.26)	98 (19.7%)
MIP [kPa]^#^	498	7.04 (4.98–9.39)	273 (54.8%)
MIP [% predicted]	499	100 (74–129)	221 (39.4%)
p0.1/MIP	498	0.03 (0.02–0.04)	354 (71.2%)
*CPET*			
VO_2max_ [ml/min]	73	1783 (1566–2195)	
VO_2max_ [% predicted]	73	93 (81–107)	
Oxygen pulse [% predicted]	73	105 (92–115)	
BR [% predicted]	73	38 (30–50)	
VE CO_2_ slope	73	28.41 (25.48–31.30)	
RER	73	1.08 (1.02–1.14)	
Peak heart rate [% predicted]	73	92 (83–98)	

*FEV*_*1*_ forced expiratory volume in one second, *FVC* forced vital capacity, *VC* vital capacity, *TLC* total lung capacity, *KCOcSB* transfer factor, *pO*_*2*_ / *pCO2* partial pressure of O_2_ / CO_2_, respectively, *SaO*_*2*_ arterial oxygen saturation, *p0.1* airway occlusion pressure, *MIP* maximum inspiratory pressure, *VO*_*2max*_ maximum oxygen uptake, *BR* breathing reserve, *VE CO*_*2*_ *slope* ventilation / CO_2_ production, *RER* respiratory exchange ratio, *IQR* interquartile range

^#^ Reference range for lung function measurements: Results were considered within the reference range if values were ≥ 80% of predicted value. Tiffeneau index (FEV1/VC) values ≥ 70% were considered physiological. Reference values for blood gas analysis: pH:7.35–7.45; pCO_2_: 35–48 mmHg; pO_2_ >83 mmHg; SaO_2_:95–99%. Reference ranges for inspiratory muscle strength measurements: p0.1 < 0.3 kPa, MIP [kPa] > 7.0 kPa for women or > 8.0 kPa for men [41], MIP (% predicted): ≥80%, p0.1/MIP < 2%.

### Inspiratory muscle strength

During 55% of visits, maximum inspiratory pressure (MIP) was considered pathologically based on cut-offs published previously [41] (Table 2). During 39.4% of visits, MIP was below 80% of the predicted normal value (calculation of predicted values based on [42]). Time since diagnosis and hospitalisation did not have a significant impact on MIP. There was a trend towards higher FAS scores in patients with MIP below reference values in linear mixed model analysis (fixed effect + 1.65 points between groups, *p* = 0.0661, median FAS [IQR] across all visits: 29 [23–37] vs. 26 [19–34]).

### Cardiopulmonary exercise testing (CPET)

For 69 patients, CPET data of sufficient quality was available from 73 tests (Table 2). In total, 45 patients reached a maximal metabolic effort, defined by a respiratory exchange ratio (RER) > 1.05 [43]. In 21 (28.8%) of all examinations, maximum oxygen uptake (VO_2max_) was below 85% of predicted values, indicating impaired cardiopulmonary exercise tolerance. Deconditioning/lack of fitness and cardiac causes were identified as probable cause most frequently, accounting for 9 (42.9% of cases with impaired exercise tolerance) each (Supplementary Table S2).

**Fig. 2:**
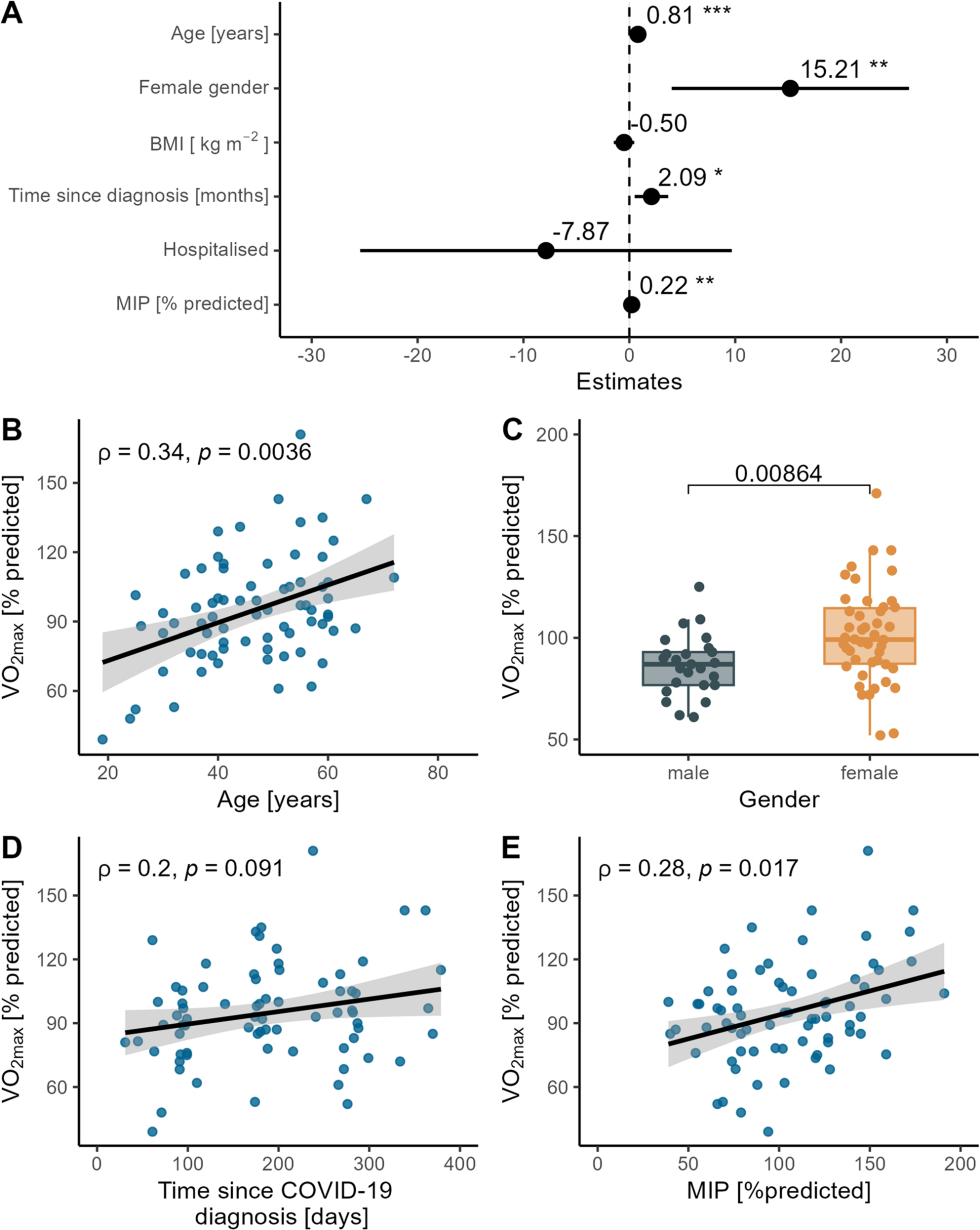
Predictors of maximum oxygen intake during CPET. **A** Results of linear mixed models (unadjusted univariable analysis) describing the fixed effects of the indicated variables on maximum oxygen uptake (VO_2max_). **B** Correlation of age of participant with outcome of VO_2max_ [% predicted] (Spearman Rank correlation). **C** Results of VO_2max_ measurements by gender. Statistical analysis was performed using linear mixed models with gender as fixed effect and patient-identifier as random effects variable. **D** Correlation of time since diagnosis of COVID-19 and VO_2max_ (Spearman Rank Correlation). **E** Correlation of MIP (% predicted) and VO_2max_ (% predicted, Spearman Rank correlation). Abbreviations: BMI: body mass index; MIP: maximum inspiratory pressure; VO_2max_: maximum oxygen intake

In univariable analysis, VO_2max_ (% of predicted normal) was found to be significantly influenced by age (linear mixed model, estimated fixed effect 0.8117/year, *p* < 0.001), gender (estimated fixed effect for female gender + 15.21%, *p* = 0.0086), time since COVID-19 diagnosis (estimated fixed effect: 2.09%/month, *p* = 0.011) and MIP (estimated fixed effect 0.22%/ % MIP, *p* = 0.0035) (Fig. 2A). In line with this, VO_2max_ correlated with age (Spearman ρ = 0.34, *p* = 0.0036), and MIP [% predicted] (Spearman ρ = 0.28, *p* = 0.017), and was higher in women (median VO_2max_ [IQR] 99% [87–114%]) than in men (median VO_2max_ [IQR] 86% [77–93%], *p* = 0.0086). Correlation of time since initial COVID-19 diagnosis and VO_2max_ failed to reach statistical significance in the present data set (Spearman ρ = 0.2, *p* = 0.091) (Fig. 2B-D).

### Serum markers

We next aimed to assess ongoing systemic inflammation, pulmonary epithelial and vascular damage as potential contributors to the presentation of long COVID-19 syndrome. Of the patient group described above, serum samples were available from 225 patients during 362 visits. Of these visits, 54 included CPET.

**Fig. 3:**
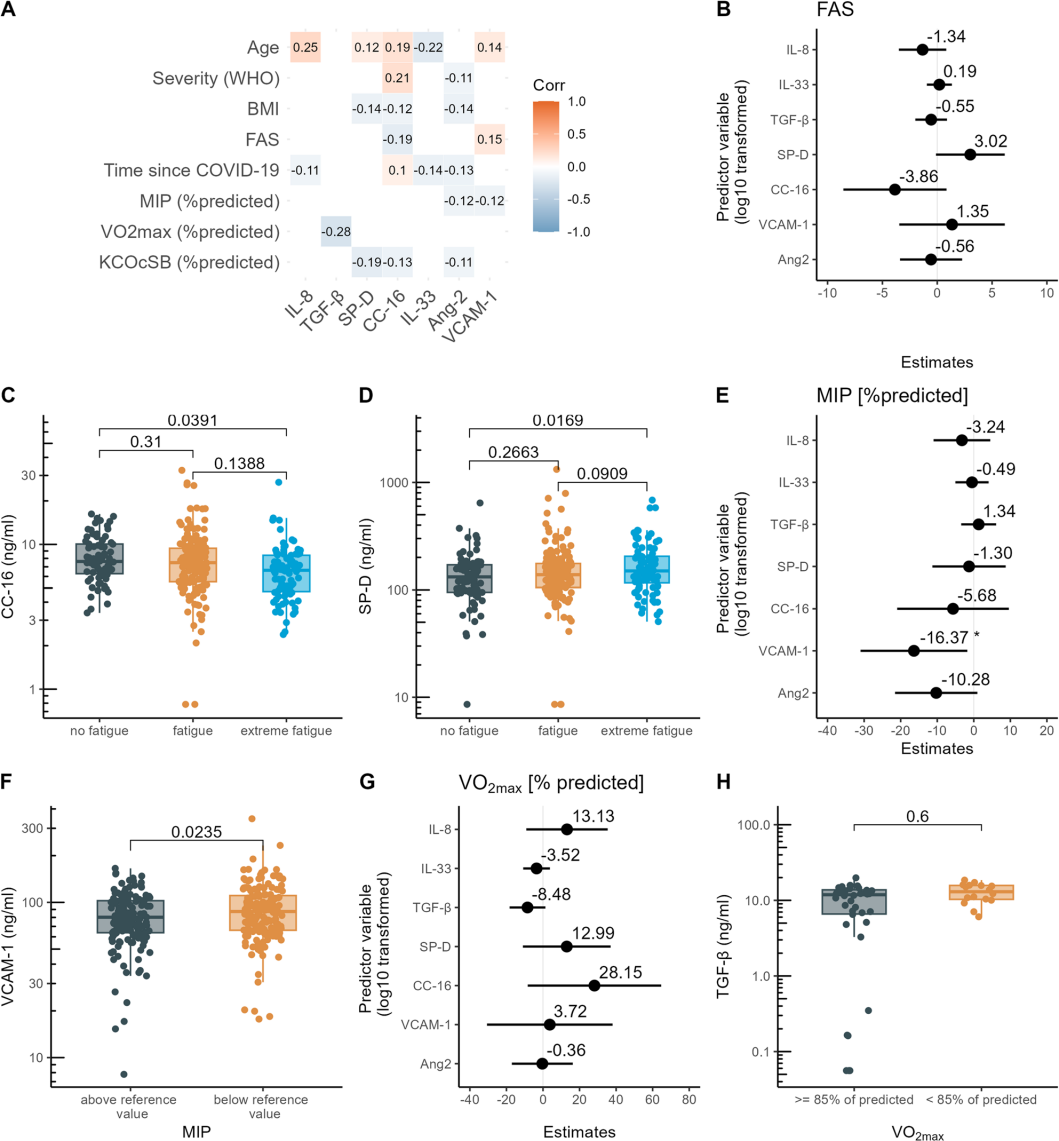
Serum markers in patients with persisting symptoms following COVID-19 and association with clinical characteristics. **A** Correlogram of Spearman Rank correlations for concentrations of serum proteins and selected demographic and clinical parameters. Values shown represent Spearman’s ρ for correlations with unadjusted *p* < 0.05. **B** Results of linear mixed models describing the fixed effects of the concentrations (log10 transformed) of indicated serum proteins on FAS scores (unadjusted univariable analysis, patient identifiers were using as random effects variable). **C** CC-16 concentrations and (**D**) SP-D concentrations in serum by fatigue severity (classified based on FAS scores). Statistical analysis was performed using linear mixed models with log10 transformed protein concentrations, fatigue category as fixed effects variable, and patient identifier as random effects variable. **E** Results of linear mixed models describing the fixed effects of the concentrations (log10 transformed) of indicated serum proteins on MIP (% predicted) (unadjusted univariable analysis, patient identifiers were using as random effects variable). **F** VCAM-1 concentrations in serum by dichotomised result of MIP measurements. Statistical analysis was performed using linear mixed models with log10 transformed protein concentrations, MIP category as fixed effects variable, and patient identifier as random effects variable. **G** Results of linear mixed models describing the fixed effects of the concentrations (log10 transformed) of indicated serum proteins on VO_2max_ (%predicted) (unadjusted univariable analysis, patient identifiers were using as random effects variable). TGF-β concentrations in serum by dichotomised result of VO_2max_ measurements. Statistical analysis was performed using linear mixed models with log10 transformed protein concentrations, VO_2max_ category as fixed effects variable, and patient identifier as random effects variable. Abbreviations: BMI: body mass index; FAS: fatigue assessment scale; MIP: maximum inspiratory pressure; VO_2max_: maximum oxygen intake; KCOcSB: transfer factor; IL-8: interleukin 8; IL-33: interleukin 33; TGF-β: transforming growth factor β; SP-D: surfactant protein D; CC-16: club cell protein 16; VCAM-1: vascular cell adhesion molecule 1; Ang2: Angiopoietin 2

In pilot measurements, concentrations of several cytokines and other proteins of interest were tested, including IL-1β, IL-6, IL-17, IL-1RA, IFN-γ, TNF-α, and ICAM-1. Measurements were below the detection limit or quantifiable range in more than 75% of samples, and the analytes were therefore not further considered in analyses (data not shown). Results of the remaining analytes are shown in Fig. 3. Considering data from all available measurements, several serum proteins were weakly correlated with relevant demographic data and outcomes (Fig. 3A). These associations were further probed using linear mixed models, to take into account patient-specific random effects. In these models, serum concentrations of CC-16 and SP-D failed to show a statistically significant effect on FAS scores Fig. 3B), however, serum levels of CC-16 were statistically significantly lower in patients with extreme fatigue (FAS score ≥ 35) than in those with no fatigue (Fig. 3C). Conversely, concentrations of SP-D were statistically significantly higher in this group when compared to those of participants without fatigue (Fig. 3D). Among the serum markers analysed, only VCAM-1 showed a statistically significant association with MIP (% predicted) in linear mixed models (Fig. 3E), and VCAM-1 levels on average were lower in individuals with MIP below reference values defined in [30] (Fig. 3F). None of the analytes tested as predictor of maximum oxygen intake during CPET (VO_2max_ [% predicted]) yielded statistically significant linear mixed models (Fig. 3G). Serum concentrations of TGF-β, which showed the highest correlation with VO_2max_ [% predicted] values in Spearman rank correlations, did not differ between individuals with maximum oxygen intake above or below the threshold of 85% predicted (Fig. 3H).

## Discussion

We here present an interim analysis of data from a prospective, observational study of patients presenting due to persistent complaints following COVID-19. Participants experienced a wide range of symptoms with a high symptom burden, most evidently fatigue, dyspnoea, and problems concentrating. For the majority of participants, lung function measurements were not indicative of restrictive or obstructive type of ventilatory impairments of lung function. However, inspiratory muscle strength was below gender-specific cut-offs in more than half of cases, and impaired cardiopulmonary exercise tolerance was observed in 28.8% of tests conducted. Explorative analyses of serum concentrations of proteins linked to inflammation, epithelial and endothelial damage and their correlation with clinical characteristics showed some weak, yet statistically significant correlations. Absolute differences between groups, however, were small. The study design moreover does not allow for mechanistic conclusions.

The range and frequency of the symptoms reported by study participants are in line with those identified by previous studies, (systematic) reviews and meta analyses [44–46], several of which have also underlined the substantial impact of COVID-19 sequelae on health-related quality of life. Even though only 15% of cases required hospitalisation during acute infection, long-term symptom burden was high. In line with our findings, an analysis by Fernández de las Peñas et al. found that the range of symptoms occurring post COVID-19 is similar in hospitalised and non-hospitalised individuals [47], providing further evidence that also mild to moderate courses of acute infection can result in significant long-term impairments of health.

More than half of participants of this study stated that fatigue, the most frequently reported symptom, affected them “strongly” or “extremely”. Based on results from the FAS questionnaire, fatigue was more common and more pronounced in women than in men and positively correlated with BMI. In agreement with this observation, female sex, and in some studies additionally high BMI/obesity, has been identify as risk factor for post COVID-19 syndrome [48–51].

Despite the high prevalence of respiratory symptoms, measurements of airflow, airway resistance, vital capacity, and lung volumes were in a physiological range for most participants and did not correlate with severity of respiratory symptoms. DLCOcSB was below 80% of predicted in 66% of measurements, however, the reduction was modest in most cases (median [IQR] DLCOcSB: 75% [67–82]). When considering alveolar volume, CO transfer (KCOcSB) was below 80% of normal in 19% of measurements. These observations are consistent with the results of several previously published meta-analyses which found reduced DLCO to be the most commonly found abnormality in lung function testing in individuals with COVID-19 sequelae [52–54], albeit with considerable heterogeneity between studies. Current data suggest that prolonged CO-diffusion disturbance may be caused by impaired pulmonary capillary circulation.

Notably, inspiratory muscle strength was below sex-specific cut offs during 55% of measurements, and 32% of measurements were below 80% of predicted values. Impaired inspiratory muscle strength in post COVID-19 syndrome has been reported previously in several studies [55–57], and consequently, respiratory muscle training programs (partially in addition to other physical training interventions) have yielded promising results in first randomised controlled trials [58, 59].

Results of CPET measurements were available for a subset of patients and revealed impaired oxygen uptake (VO_2max_) during exercise in 28.8% of measurements, most of which was likely to be attributable to deconditioning or cardiac causes (43% each of cases with impaired exercise tolerance). Various studies have described reduced exercise capacity following infection with SARS-CoV-2 [19, 55, 60–66], although there is considerable heterogeneity between studies. The use of different cut offs, risk of sampling bias and consequently differences in characteristics of the study populations are likely to contribute to the differences in observations. Similar to our findings, exercise intolerance did not correlate with symptoms or degree of post COVID-19 disability in other studies [60, 67]. In agreement with the data presented here, deconditioning was frequently identified as a major underlying cause by Durstenfeld et al. [62].

Persistent inflammation and aberrant activation of immune cells have been hypothesised to contribute to the development of post COVID-19 syndrome. Multiple studies found circulating levels of certain cytokines to be higher in patients with post COVID-19 syndrome than in control groups, and some studies identified aberrant patterns of immune cell activation in patients with post COVID-19 syndrome [11, 16, 68]. However, in another cohort, immunological aberrances in post COVID-19 syndrome appeared to be restricted to the lung and were not evident in the blood of study participants [12]. A meta-analysis published in 2023 found levels of CRP, D-dimer, LDH and leukocytes to be significantly higher in COVID-19 survivors with post COVID-19 syndrome than in controls [69]. Inflammatory cytokines were included only in fewer studies and no significant association with the occurrence of post COVID-19 syndrome was found for these analytes in this meta-analysis. In our study, several pro-inflammatory cytokines described as elevated in post COVID-19 syndrome in other studies were below the quantifiable range in most samples analysed, despite the use of sensitive assays. This suggests that the present study population was not characterised by a generalised systemic inflammatory state. This difference in circulating levels of inflammatory markers compared to previously published studies might be due to differences in the time elapsed since the acute infection as well as the severity of acute infection. In our analyses, we found only weak correlations of those serum markers that were quantifiable with clinical characteristics, including lung function and symptom scores. Whether these are linked to pathophysiological mechanisms remains to be investigated. We observed small, yet statistically significant differences in the concentrations of CC-16 and SP-D in patients with extreme fatigue compared with participants without fatigue: Whereas serum concentrations of CC-16 were on average lower in the group with severe fatigue, levels of serum SP-D were on average higher in serum samples of these individuals. CC-16 has been described previously as a marker for epithelial damage, but also possesses anti-inflammatory and protective effects properties (reviewed in [70]). Elevated levels of SP-D likewise can be indicative of pulmonary damage, and have additionally been found to be associated with impaired diffusion capacity in patients with persistent dyspnoea after COVID-19 [71]. Considering the small magnitude of the differences and large variation in the study population described here, the biological implications of these findings remain unclear. Similarly, also biomarkers of the endothelium showed only limited to no association with FAS scores or lung function parameters. The analyses presented here therefore remain explorative and do not allow for mechanistic conclusions.

### Limitations and strengths

Limitations of the analysis presented here include the monocentric design of the study and the lack of a control group. The observations made in this group might therefore be non-specific to post COVID-19 syndrome. Since data were obtained during clinical routine visits of patients attending a specialised outpatient clinic, the observed improvements over time might underestimate the actual effects, as patients who are no longer experiencing symptoms frequently do not present for additional examinations.

At the start of the study, there was a lack of validated questionnaires for patients with or recovering from COVID 19. The questionnaires used were not specifically developed for post-COVID-19 syndrome and may not have detected all symptoms with sufficient validity. Furthermore, neurosensory complaints, such as anosmia and ageusia, were not included in the structured symptom assessment, even though they may define a relevant clinical phenotype of individuals with post-COVID-19 syndrome [72].

The majority of cases included in this analysis had a mild to moderate course of acute illness and did not require hospitalisation during acute COVID-19. These numbers are similar to the findings of Dennis et al., who found that 84% of patients with severe post COVID-19 syndrome hat not been hospitalised [73]. Moreover, no pre-selection was made regarding severity of acute COVID-19 or manifestation of post COVID-19 syndrome. The results therefore represent real-life data capturing the heterogeneity and complexity of post COVID-19 syndrome. On the other hand, we must be aware of a selection bias because only symptomatic patients were enrolled in the study, which was conducted in an outpatient clinic. Patients included in this analysis were infected early during the pandemic, encompassing the time between beginning of the pandemic until May 2021, corresponding to the first three pandemic waves in Germany [40]. At this time, the dominant variants circulating in central Europe were wildtype, alpha, and beta. The first vaccines against SARS-CoV-2 became available in Germany at the end of December 2020 with an initial prioritisation of vulnerable groups and healthcare workers, therefore most patients in this analysis were unvaccinated at the time of infection. Vaccination prior to infection as well as after infection appears to reduce the risk to develop post COVID-19 syndrome, and is suggested to reduce some of the symptoms commonly found in post COVID-19 syndrome [74, 75]. Lack of immunity, along with higher virulence of the first circulating variants might increase the risk to develop post COVID-19 syndrome, and future analyses will need to evaluate the impact of vaccinations and variant-specific effects in post COVID-19 syndrome.

CPET and pulmonary function testing was performed according to the local clinical standard. Therefore, the CPET protocol was chosen by the involved physician which makes comparison with other data difficult. Another shortcoming is that not all patients underwent exercise testing. Finally, the statistical analyses were performed using univariate and linear mixed models and therefore do not take into account confounding factors.

### Conclusions and outlook

The present study confirms the high and persistent symptom burden as well as the marked heterogeneity of post-COVID manifestations. While conventional spirometric indices were largely preserved, impairments in inspiratory muscle strength and exercise capacity were frequently observed. Nevertheless, associations between clinical symptoms, physiological measures, and circulating biomarkers were weak, underscoring the limited explanatory or predictive value of isolated diagnostic modalities. These findings support the concept of heterogeneous post-COVID phenotypes, potentially driven by distinct pathophysiological mechanisms. Future studies should focus on integrated phenotyping approaches that account for functional limitations, comorbidities, and biomarker profiles, as well as on the differentiation from other post-infectious syndromes, to improve risk stratification and guide targeted management strategies.

## Supplementary Information

Supplementary Material 1.

## Data Availability

The datasets used and analysed during the current study are available from the corresponding author on reasonable request and if legal requirements regarding data protection can be met.
