# Supplementary material for: Symptom burden, lung function, exercise tolerance and inflammation in patients post COVID-19: results from the prospective COVID-19 Chronic Morbidity (CCHROMO) study
Source: BMC Pulm Med. 2026 Jun 17;26:272. doi: 10.1186/s12890-026-04365-1 (PMC13274024; doi:10.1186/s12890-026-04365-1)
Supplement: Supplementary file 1 — Supplementary Material 1. [file 12890_2026_4365_MOESM1_ESM.pdf]

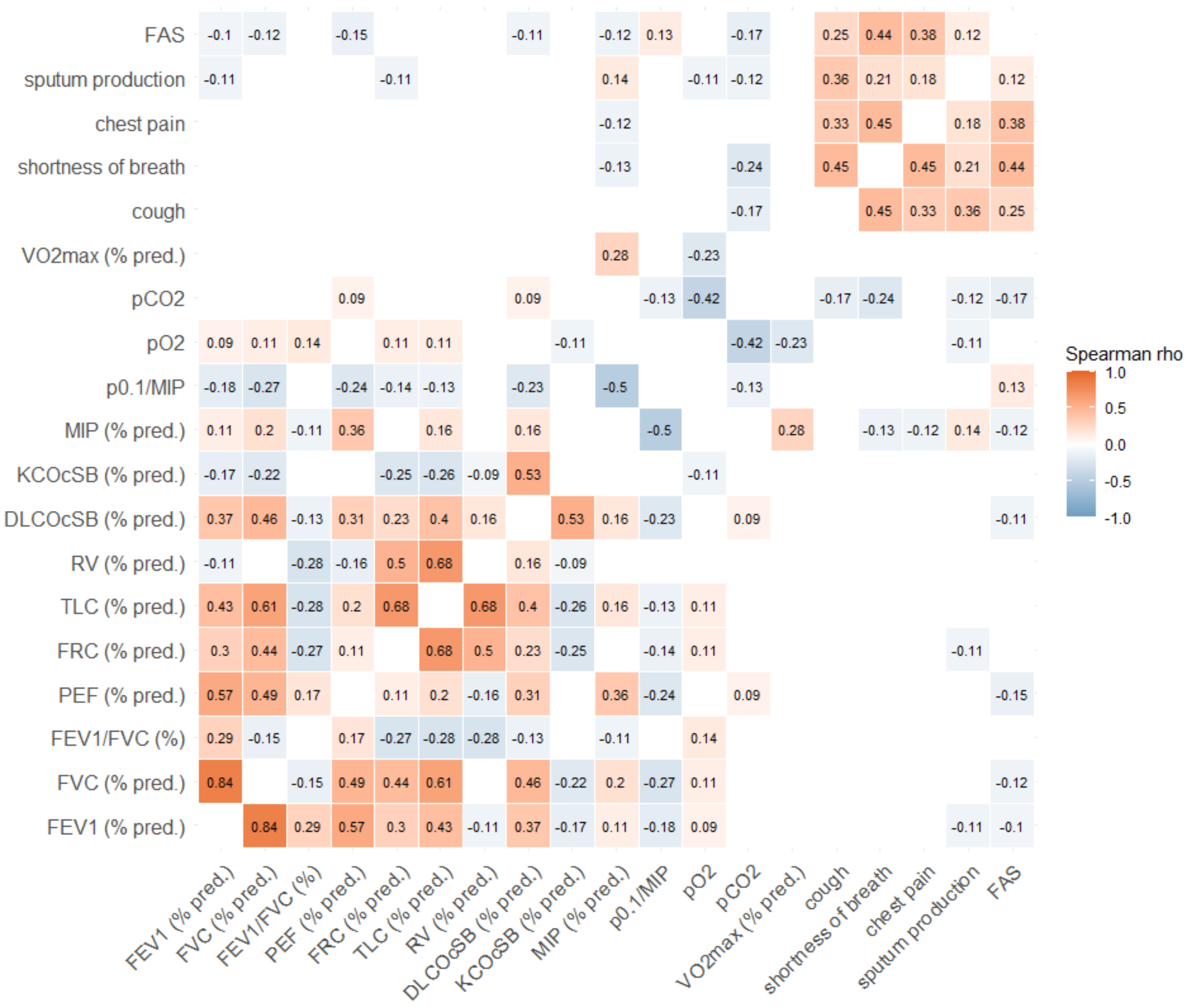

Supplemental Figure 1. Correlogram of lung function parameters with symptom scores. Spearman rank correlations were calculated for lung function parameters, fatigue scores and respiratory symptom scores as assessed as part of the CAP18 questionnaire and FAS. Correlogram shows significant correlations ($p<0.05$) and respective Spearman ρ. Abbreviations:FEV1: forced expiratory volume in 1 second; % pred.: % of predicted normal; FVC: forced vital capacity; PEF: peak expiratory flow; FRC: functional residual capacity; TLC: total lung capacity; RV: residual volume; DLCOcSB: single breath carbon monoxide uptake (corrected); KCOcSB: transfer factor; MIP: maximum inspiratory pressure; p0.1: airway occlusion pressure; pO_2 / pCO2: partial pressure of O_2 / CO_2, respectively; VO_{2max}: maximum oxygen intake; FAS: fatigue assessment scale

Supplementary Table S1. Additional lung function and CPET parameters. Abbreviations: PEF: peak expiratory flow; FRC; functional residual capacity; RV: residual volume; DLCOcSB: single breath carbon monoxide uptake (corrected);

Variable	N	Median (IQR)	Outside reference range n (%)
Lung function			
PEF [% predicted]	558	104 (89-119)	82 (14.7 %)
FRC [% predicted]	544	101 (87-118)	72 (13.2 %)
RV [% predicted]	540	101 (84-120)	107 (19.8 %)
DLCOcSB [% predicted]	533	75 (67-82)	354 (66.4 %)
CPET			
Workload [% predicted]	73	109 (93-137)	
Aerobic capacity [(ml/min) / W]	73	10.26 (9.53-11.06)	

Reference ranges for inspiratory muscle strength measurements: p0.1 < 0.3 kPa, MIP (kPa) >7.0 kPa for women or >8.0 kPa for men [1]

Supplementary Table S2. Probable causes for exercise intolerance in patients with post-COVID-19 syndrome undergoing cardiopulmonary exercise testing (CPET). V/Q mismatch: ventilation-perfusion mismatch, VO_{2max}: maximal oxygen uptake, BR: breathing reserve (% predicted), VT1 (% predicted): first ventilatory threshold (% predicted), VE CO_2: exhaled carbon dioxide slope.

		All CPETs	CPETs with full metabolic effort (RER > 1.05)
	Criteria	n (% of all CPETs)	n (% of all CPETs with full metabolic effort)
Total	/	73	45
Impaired cardiopulmonary exercise tolerance	VO_{2max} < 85% predicted	21 (28.8 % of all CPETs)	12 (26.7 %)
Probable cause for impaired exercise tolerance		n (% of patients with VO_2max < 85%)	n (% of patients with VO_2max < 85%)
Pulmonary	VO_{2max} < 85 % predicted & BR <= 15 %	0 (0 %)	0 (0 %)
Cardiac	VO_{2max} < 85 % predicted & oxygen pulse ≤ 80 % predicted	9 (42.9 %)	5 (41.7 %)
Deconditioning / lack of fitness	VO_{2max} < 85 % predicted & $VT1_{max}$ ≤ 40 % & oxygen pulse ≥ 80 % predicted & BR > 15 % & VE CO_2 slope < 34	9 (42.9 %)	5 (41.7 %)
V/Q mismatch	VO_{2max} < 85% predicted & $VT1_{max}$ > 40 % & oxygen pulse > 80 % predicted & BR > 15 % & $VECO_2$ slope ≥ 34	1 (4.8 %)	1 (8.3 %)
Unclear		2 (9.5 %)	1 (8.3 %)
